# Epidemiology and Genomic Characterization of *Trichophyton mentagrophytes* over a Period of 4 Years in Northern Italy

**DOI:** 10.3390/jof11080566

**Published:** 2025-07-29

**Authors:** Luca Rossi, Annarita Sorrentino, Caterina Signoretto, Paolo Gaibani

**Affiliations:** 1 Microbiology Section, Department of Diagnostic and Public Health, Verona University, 37134 Verona, Italy; dr.rossiluca@gmail.com (L.R.); caterina.signoretto@univr.it (C.S.); 2 Microbiology and Virology Unit, Azienda Ospedaliera Universitaria Integrata di Verona, 37134 Verona, Italy; annarita.sorrentino@aovr.veneto.it

**Keywords:** dermatophytes, *Trichophyton mentagrophytes*, genotype I/II, terbinafine resistance, *SQLE* gene, whole-genome sequencing

## Abstract

Dermatophytes are keratinophilic fungi that cause a wide range of superficial infections in humans and animals. The *Trichophyton mentagrophytes* species complex is one of the most clinically important groups due to its broad host range, widespread distribution, and increasing involvement in antifungal-resistant infections. Here, we described the epidemiology of *T. mentagrophytes* over a period of 4 years detected in the northeastern part of Italy and provided the genomic characterization of clinical isolates. ITS sequence analysis revealed that among the 13 strains studied, 11 belonged to the *T. mentagrophytes* complex. In detail, nine were classified as genotype I/II and two as genotype VII. Analysis of the *SQLE* gene revealed that nine strains harbored a wild-type gene, while two carried a Lys276Asn mutation. Genomic analysis was performed on three clinical *T. mentagrophytes* strains that belonged to genotype I/II, revealing the presence of different virulence factors including MEP-1, MEP-2, MEP-3, and MEP-5. Phylogenetic analysis based on core-genome SNPs demonstrated that the two genomes included in this study were clonally related to a *T. mentagrophytes* strain isolated in China in 2024. In conclusion, our study highlights the importance of genomic characterization in order to trace the epidemiology of dermatophytes worldwide and to characterize emerging strains.

## 1. Introduction

In the last few decades, antifungal resistance has become an expanding and increasingly significant issue [1]. Pathogenic fungi, responsible for both invasive and superficial infections, are progressively developing resistance to various classes of antifungal drugs [2].

Dermatophytes, a group of keratinophilic fungi, are among the most common causative agents of superficial fungal infections [3,4]. Among different species, the most common etiological agents of dermatophytosis in human are *Trichophyton rubrum*, *Trichophyton interdigitale* and *Trichophyton mentagrophytes*, which are able to colonize keratinized tissues such as skin, hair, and nails [5,6].

Recently, emergence cases of *T. mentagrophytes* resistant to antifungal molecules have been reported in different countries [2,7]. In particular, different cases of dermatophytosis infections due to terbinafine-resistant *T. mentagrophytes* were reported in India. In this context, genotypic analysis demonstrated that clinical isolates belonged to genotype VIII, which was further named as *T*. *indotineae* by Kano et al. [2,7,8].

In the last few years, *T*. *indotineae* has become a leading cause of widespread and difficult-to-treat skin infections especially in India, where it has driven an epidemic of extensive dermatophytosis [1,9]. In particular, epidemiological and molecular studies have shown that *T. indotineae* initially spread in Asia, showing a gradually replacing *Trichophyton rubrum* as the predominant endemic dermatophyte. Currently, it has become the most widespread dermatophyte in different countries [1,2,3,4,5,6,7,8,9]. Due to its microscopic and macroscopic similarity to other species of the *T. mentagrophytes* complex, accurately identifying *T. indotineae* requires advanced molecular tests, such as the sequencing of ITS regions. *T. indotineae* exhibits commonly resistance to terbinafine, which is a first-line topical and systemic antifungal agent used to treat dermatophytic infections. It acts by inhibiting the enzyme squalene epoxidase, disrupting ergosterol biosynthesis, a vital component of fungal cell membranes, and leading to the toxic intracellular accumulation of squalene [9,10]. Although different studies reported the enlarging widespread of *T. indotinae* in different continents, few epidemiological data are available in Italy [1,2,3,4,5,6,7,8,9].

Based on these considerations, the aim of the present study was to characterize the *T. mentagrophytes* strains collected in a hospital located in the northeastern part of Italy over a period of 4 years and to evaluate the susceptibility to terbinafine and the related genetic mechanism of resistance. At the same time, we characterized the genomes of different clinical strains, the object of this study, in order to enlarge the knowledge of the *T. mentagrophytes* epidemiology.

## 2. Material and Methods

### 2.1. Clinical Isolates

Between 1 January 2020 and 31 December 2024, we collected all fungal strains from clinical samples of nail scales, skin scales, and hair from patients with superficial mycosis who were admitted to a large tertiary hospital located in the northeastern part of Italy. Species identification was initially performed by fungal culture and based on morphological examination of the colonies and following microscopic examination morphological characteristics of the fungal colonies following the routine workflow.

### 2.2. Evaluation of Terbinafine Sensitivity

To evaluate the sensitivity of the studied strains to terbinafine, the TCAM (Terbinafine Containing Agar Method) was used, which involves Sabouraud Dextrose Agar (SDA) enriched with terbinafine at a concentration of 0.2 mg/L [5,11]. The medium was prepared following these steps: 500 mL of distilled water was measured using a cylinder, and 15 g of Sabouraud Dextrose Liquid Medium (Thermo Scientific™ Oxoid™ Sabouraud Dextrose Broth, Waltham, MA, USA) was added to the water along with 7.5 g of Bacteriological Agar Type E (Microbiol Diagnostici, Macchiareddu, Italy). The mixture was sterilized in an autoclave at 121 °C for 15 min. After sterilization, the medium was allowed to cool to 50–60 °C, and 15 mL was poured into each Petri dish. Half of the plates were enriched with terbinafine to achieve a final concentration of 0.2 mg/L, while the other half were left as growth controls. Each strain was inoculated on both a TCAM plate and an SDA control plate without terbinafine and incubated at room temperature. Growth was evaluated after 7 and 14 days [5,11].

### 2.3. Molecular Identification of ITS Region and SQLE Gene of Clinical Isolates

DNA from the fungal colonies was extracted according to the manufacturer’s protocol using the InGenius system (Elitech, Signes, France). The identification of isolates from the *T. mentagrophytes* complex was performed at both the species and genotype level by PCR sequencing of the ribosomal DNA (rDNA) internal transcribed spacer (ITS), as previously described [12,13]. Terbinafine resistance was investigated by amplification of the *SQLE* gene by using TrSQLE-F1 (5′-ATGGTTGTAGAGGCTCCTCCC-3′) and TrSQLE-R1 (5′-CTAGCTTTGAAGTTCGGCAAA-3′) primers and related amplification settings as previously described [12]. The amplification products of the ITS regions and *SQLE* gene were sequenced using the Sanger method, and multiple sequence alignment was performed with ClustalOmega v1.2.4 and visualized with Unipro UGENE v49.1. Phylogenetic analysis was performed with MEGA v11.0.11 (available at: https://www.megasoftware.net; accessed on 10 January 2025) using the iterative Neighbor-Joining (NJ) algorithm based on the distance matrix. The list of *T. mentagrophytes* strains used for phylogenetic analysis based on the ITS sequence is shown in Table 1.

### 2.4. Genomic Characterization of Clinical Isolates

Genomic DNA extraction, purification, quantification and library preparation were performed as previously described [13]. Briefly, DNA sequencing was conducted on the Illumina MiSeq system (Illumina, San Diego, CA, USA). The obtained paired-end reads underwent quality control with FastQC v0.12.1 (available at: https://www.bioinformatics.babraham.ac.uk/projects/fastqc/; accessed on 10 January 2025), and adapters were trimmed with Trimmomatic v0.39 (available at: https://github.com/usadellab/Trimmomatic; accessed on 10 January 2025). Prior to proceeding with the genome assembly, reads were filtered to remove any contaminant sequences, as previously described [14]. Briefly, Illumina reads were mapped against the reference genome of the *T. mentagrophytes* ATCC18748 (Acc. no. BPUJ00000000.1) using Burrows–Wheeler Aligner (BWA) v.0.7.18 software. Mapped reads were extracted with Samtools 1.6 and used for the de novo genome assembly with SPAdes v4.0.0 using careful settings. Assemblies were annotated by using AUGUSTUS (available at: https://bioinf.uni-greifswald.de/augustus/; accessed on 10 January 2025), and gene prediction was manually investigated by BLAST analysis. Genes involved in virulence were detected using the PHI-base (Pathogen-Host Interaction) database by BLAST analysis.

The *T. mentagrophytes* genomes available in GenBank and used for the molecular epidemiological analysis are shown in Table 2. A phylogenetic tree based on core genome single nucleotide polymorphisms (SNPs) was performed using ParSNP software (available at: https://harvest.readthedocs.io/en/latest/content/parsnp.html; accessed on 10 January 2025) using the genome of *T. mentagrophytes* ATCC 18748 as a reference. In addition, ParSNP analysis was performed using the settings “-c”, “-a 13” and “-x” for all of the genomes, higher resolution mapping and the exclusion of SNPs located in regions of recombination. A maximum likelihood tree was constructed from the final alignment of core-genome SNPs using FastTree with generalized time-reversible mode.

## 3. Results

### 3.1. Phenotypic and Genotypic Characterization of Dermatophytes Clinical Strains

**Figure 1:**
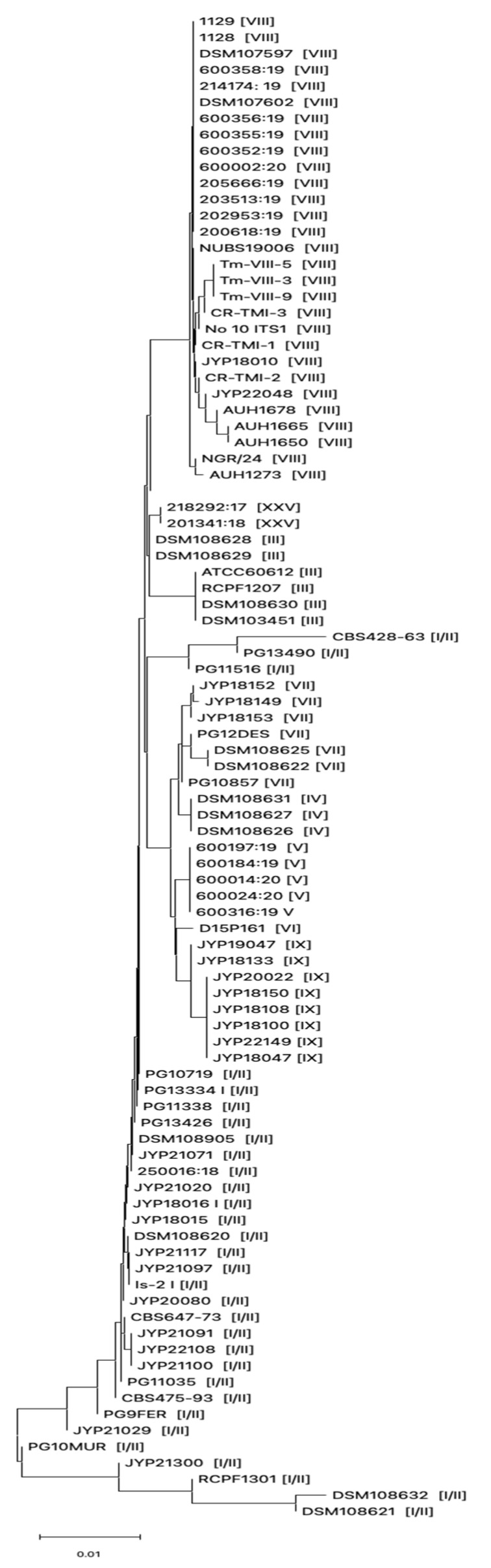
Evolutionary history inferred using Neighbor-Joining method based on ITS sequences derived from *T. mentagrophytes* strains collected in this study and from different countries. Genotype for each strain is shown within the brackets.

During the study period, we isolated a total of 13 dermatophytes collected from different types of skin samples by culturing and macro and microscopic examination. The list of strains included in this study is shown in Table 3. In order to characterize genotypically the dermatophytes isolates included in this study and due to the impossibility of distinguishing between genotypes belonging to the *T. mentagrophytes* complex, the ITS rDNA were sequenced and compared to ITS regions derived from *T. mentagrophytes* strains belonging to different genotypes present in GenBank (Table 1). ITS analysis demonstrated that 1 out of 13 dermatophytes belonged to the *T. verrucosum* species, while 11 out of 13 clinical strains included in this study belonged to the *T. mentagrophytes* complex. *Beauveria bassiana* was excluded from the study by not belonging to the dermatophytes. Phylogenetic analysis based on the ITS region of the 11 isolates genotypically characterized as *T. mentagrophytes* showed that 9 (81.8%) strains belonged to genotype I/II, while 2 (18.2%) belonged to genotype VII (Figure 1).

Antifungal susceptibility testing results showed that all *T. mentagrophytes* strains included in this study were susceptible to terbinafine.

Genotypic characterization revealed that 9 out of 11 *T. mentagrophytes* clinical strains harbored the wild-type *SQLE* gene, while two strains harbored a Lysin for Asparagine substitution at position 276 (Table 3). Correlation with ITS genotypic results revealed that strains harboring the Lys276Asn substitution belonged to the *T. mentagrophytes* genotype I/II.

### 3.2. Genomic Characterization of T. mentagrophytes Genotype I/II

In order to characterize the *T. mentagrophytes* isolates included in this study, we performed the whole-genome sequencing (WGS) of selected strains included in this study (PG9FER, PG10MUR and PG11338).

WGS produced a total of 18,518,774, 13,421,006 and 13,838,658 Illumina reads 300 bp in length, respectively, for PG9FER, PG10MUR and PG11338 clinical strains.

Genome assemblies of PG9FER, PG10MUR and PG11338 generated, respectively, a total of 615, 679 and 712 contigs with a G + C content of 47.98, 48 and 47.52. The characteristics of the *T. mentagrophytes* genomes included in this study are shown in Table 4. Assemblies gave a total of 7.1924 (PG9FER), 7.140 (PG10MUR) and 7.140 (PG11338) of protein-coding DNA sequences (CDSs), thus showing a similar number of CDSs observed in the available *T. mentagrophytes* genomes previously annotated (Table 2).

A genomic analysis of *T. mentagrophytes* genomes obtained in this study showed that different virulence factors (i.e., metalloproteinase, *MEP* genes) were present (Figure S1 in the Supplementary Materials). In detail, the following virulence factors (MEP-1, MEP-2, MEP-3 and MEP-5) were present in the genomes of PG9FER, PG10MUR and PG11338, while MEP-4 was present in the complete form only in the PG9FER strain, and no isolates harbored complete ZAF-A (Table 5).

**Figure 2:**
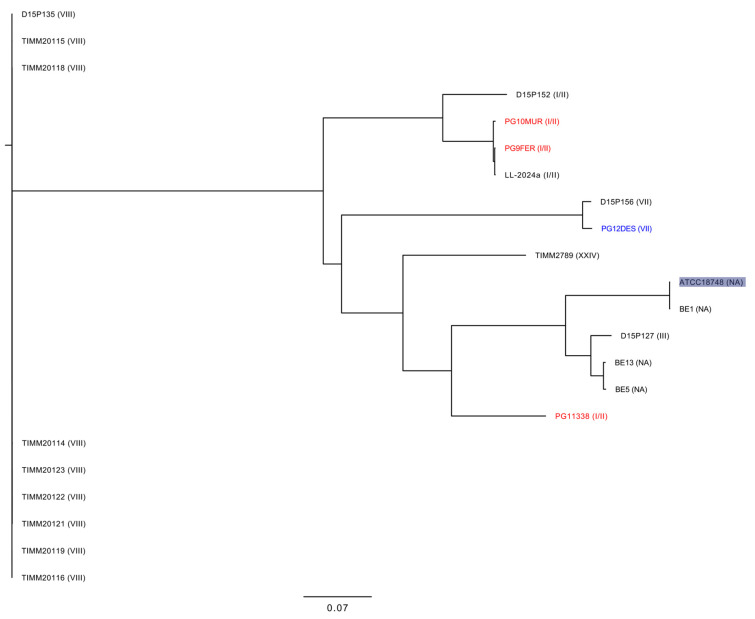
Maximum likelihood phylogeny based on SNPs in the core genomes of *T. mentagrophytes*. The *T. mentagrophytes* clinical isolates included in this study are highlighted in red, while *T. mentagrophytes* belonging to genotype VII recently isolated in Italy were highlighted in blue. The genotype for each strain is shown within the brackets. Abbreviations: NA, Not applicable.

To investigate the clonal relatedness of *T. mentagrophytes* collected in our study with the clinical available genomes isolated worldwide, phylogenetic analysis based on core genome SNPs was performed. A list of the selected genomes collected in this study and the *T. mentagrophytes* genomes available in GenBank and used for the genomic comparison is shown in Table 2. A phylogenetic tree demonstrated that all *T. mentagrophytes* genomes included in this study grouped into a monophyletic clade formed by isolates belonging to different genotypes, while *T. mentagrophytes* genomes belonging to genotype VIII (defined as *T. indotinae*) clustered separately (Figure 2). In detail, PG9FER and PG10MUR clustered closely to the LL-2024a clinical isolate, which was a strain isolated in China during 2024 from a patient with skin lesions. At the same time, PG11338 clustered separately to the genomes of PG9FER and PG10MUR strains, thus demonstrating a divergent evolution of strains belonging to this genotype. Of note, our phylogenetic analysis based on core-genome SNPs (Figure 2) revealed that the PG12DES strain, which belongs to the emergent genotype VII, was closely related to the D15P156 (NCBI GenBank GCA_003664385.1), which is a *T. mentagrophytes* strain collected in Moldova in 2017 from a patient suffering from tinea capitis who had contact with a rabbit.

A deep genome comparison with ATCC18748 and clonally related genomes (i.e., D15P152, LL-2024a) showed that PG9FER, used as a reference for comparison, exhibited OrthoANIu values of 98.2% (PG9FER vs. ATCC18748), 99.6% (PG9FER vs. D15P152), 99.94% (PG9FER vs. LL-2024a), 99.92% (PG9FER vs. PG10MUR) and 98.99% (PG9FER vs. PG11338) with an average aligned length (bp) of 16,812,337 bp. At the same time, genome comparison analysis showed that PG12DES differed by 4141 SNPs in comparison, respectively, to the D15P156 strain and showed an OrthoANIu value of 99.83% with an average aligned length (bp) of 17,009,500 bp.

## 4. Discussion

In this study, we described the epidemiology of *T. mentagrophytes* strains collected in a hospital located in the northeastern part of Italy over a period of 4 years. Our results demonstrated that the *T. mentagrophytes* included in this study belonged to genotypes I/II and VII, thus showing the endemic circulation of few genotypes among patients in the northeastern part of Italy. Although the number of patients included in this study was small, our results are in agreement with a previous study showing that genotype I/II was the most prevalent genotypes distributed worldwide [15], while *T. mentagrophytes* type VII (TMVII) was a recently emerged dermatophyte causing different outbreaks in different countries in Europe (i.e., Germany and France) and more recently in the USA [16,17,18,19]. This emerging genotype is closely related to *T. indotineae*, and it has been hypothesized to be transmitted from human to human through sexual contact with an increasing risk of infections among groups engaging in high-risk sexual behaviors, such as men who have sex with men (MSM) [16,17,18,19]. However, the PG12DES strain belonging to genotype VII was isolated from a clinical sample of skin scale located around the eyes of a young child in 2022, thus suggesting a larger spreading of genotype VII worldwide. Based on these findings, our data reinforced the importance of monitoring the spread of clinical strains in order to provide a complete picture *T. mentagrophytes* epidemiology and to characterize emerging genotypes worldwide. In particular, further genomic and/or genotypic studies need to be perform to better define the epidemiological diffusion of different genotypes among *T. mentagrophytes* in Italy.

Antifungal susceptibility analysis demonstrated that all strains were susceptible to terbinafine, and no mutations within the *SQLE* gene were observed in strains included in this study. In this context, the resistance to terbinafine diminishes the efficacy of available treatments, complicating clinical management and posing significant challenges for healthcare systems. Indeed, resistant fungal infections often lead to prolonged treatment durations, higher healthcare costs, and an increased risk of complications, underscoring their impact on public health [2].

The spread of antifungal resistance represents an escalating global health concern, thus limiting the therapeutic options available and made the management of fungal infections increasingly complex [1]. Indeed, the resistance has significant implications, requiring extended and more complex treatment regimens, which impact healthcare systems and the quality of life of affected patients.

Lastly, here, we provided the complete genome analysis of three *T. mentagrophytes* strains belonging to type I/II. Our results demonstrated that different virulence factors were conserved among our isolates (MEP-1, MEP-2, MEP-3 and MEP5), while ZafA was truncated in all strains. Genomic phylogenetic analysis revealed that the genomes of *T. mentagrophytes* included in this study were closely related to a clinical strain recently isolated in China, thus suggesting a high conservation degree of this genotype among dermatophytes. The high nucleotide homology of our strains with isolates collected from different countries indicated a high circulation of clonally related strains worldwide, requiring further genomic investigations

In conclusion, our study aims to provide a picture of the epidemiology of dermatophytosis in the northern part of Italy by providing a deep genome analysis of the common genotypes circulating in our region. Our study highlights the importance of monitoring the spread of dermatophytes in order to provide a complete view of the *T. mentagrophytes* epidemiology and to rapidly identify new genotypes or novel traits of resistance.

## Figures and Tables

**Table 1:** List of *T. mentagrophytes* strains used for the phylogenic analysis based on the ITS sequences.

Strain	Country	Host	Genotype	Acc. No.
JYP22149	China	Human	IX	OP961423
JYP18047	China	Human	IX	OP961397
JYP18108	China	Human	IX	OP961399
JYP18150	China	Human	IX	OP961402
JYP18100	China	Human	IX	OP961398
JYP20022	China	Human	IX	OP961406
JYP18133	China	Human	IX	OP961400
JYP19047	China	Human	IX	OP961405
D15P161	Russia	Cat	VI	MK722518
DSM 108631	Germany	Human	IV	MK447607
DSM 108627	Germany	Human	IV	MK447608
DSM 108626	Germany	Human	IV	MK447609
DSM 108625	Germany	Human	VII	MK450323
DSM 108622	Germany	Human	VII	MK450322
JYP18149	China	Human	VII	OP961401
JYP18153	China	Human	VII	OP961404
600316:9	Iraq	Human	V	MT374257
600024:20	Iraq	Human	V	MT374269
600197:19	Iraq	Human	V	MT374258
600184:19	Iran	Human	V	MT374259
ATCC60612	N.A.	N.A.	III	KJ606099
RCPF 1207	Russia	Human	III	KT253559
DSM 108630	Swiss	Human	III	MK450325
DSM 103451	Swiss	Leopard	III	KX866689
DSM 108632	Germany	Human	I/II	MK447606
218292:17	Cambodia	Human	XXV	MN886815
201341:18	Cambodia	Human	XXV	MN886816
DSM 108628	Germany	Human	III	MK447605
DSM 108629	Germany	Human	III	MK447604
CR-TMI-1	France	Human	VIII	MW898018
Tm-VIII-5	France	Human	VIII	MW959757
Tm-VIII-3	France	Human	VIII	MW959755
Tm-VIII-9	France	Human	VIII	MW959756
CR-TMI-3	France	Human	VIII	MW898020
No_10_ITS1	Vietnam	Human	VIII	OM108103
AUH1273	Greece	Human	VIII	MW752105
JYP22048	India	Human	VIII	OP961421
JYP18010	India	Human	VIII	OP961393
CR-TMI-2	France	Human	VIII	MW898019
AUH1678	Greece	Human	VIII	MW752108
AUH1665	Greece	Human	VIII	MW752107
AUH1650	Greece	Human	VIII	MW752111
NUBS19006	Japan	Human	VIII	LC508024
200618:19	Germany	Human	VIII	MT330285
202953:19	Germany	Human	VIII	MT330284
203513:19	Germany	Human	VIII	MT330280
600002:20	Germany	Human	VIII	MT333227
600352:19	Poland	Human	VIII	OM951136
600355:19	Poland	Human	VIII	OM951134
600356:19	Poland	Human	VIII	OM951138
214174:19	Germany	Human	VIII	MT330289
600358:19	Poland	Human	VIII	OM951141
DSM 107597	India	Human	VIII	MH791420
DSM 107602	India	Human	VIII	MH791425
1129	France	Human	VIII	ON528187
1128	France	Human	VIII	ON528186
JYP20080	China	Human	I/II	OP961407
250016:18	Cambodia	Human	II	QKE51044
DSM 108905	Germany	Human	II	QFR36155
JYP21071	China	Human	I/II	OP961415
JYP21117	China	Human	I/II	OP961419
JYP21097	China	Human	I/II	OP961417
Is-2	Tunisia	N.A.	I	KC595991
RCPF 1301	Russia	Human	I/II	AKH90742
DSM 108621	Germany	Human	I/II	QBA85641
JYP18016	China	Human	I/II	OP961395
DSM 108620	Germany	Human	I	QBA85640
JYP21020	China	Human	I/II	OP961411
JYP22108	China	Human	I/II	OP961422
JYP21100	China	Human	I/II	OP961418
JYP21091	China	Human	I/II	OP961416
JYP18015	China	Human	I/II	OP961394
JYP21029	China	Human	I/II	OP961412
JYP21300	China	Human	I/II	OP961420
CBS475.93	N.A.	N.A.	I/II	MF926357
CBS647.73	Germany	Human	I/II	KT155955
CBS428.63	N.A.	N.A.	I/II	NR_144900

Abbreviations: N.A. Not available.

**Table 2:** List of the *T. mentagrophytes* genomes used for the phylogenomic analysis.

Strain	Acc. No.	Country	Genotype
ATCC18748	GCA_045862495.1	Reference	NA
D15P127	GCA_003664465.1	Russia	III
D15P135	GCA_003664455.1	India	VIII
D15P152	GCA_003664425.1	Russia	I/II
D15P156	GCA_003664385.1	Moldova	VI
TIMM2789	GCA_003118255.1	Japan	XXIV
TIMM20114	GCA_023065905.1	India	VIII
TIMM20115	GCA_023065845.1	India	VIII
TIMM20116	GCA_023065885.1	India	VIII
TIMM20118	GCA_023065865.1	India	VIII
TIMM20119	GCA_023065815.1	India	VIII
TIMM20121	JAUJAB010000000	India	VIII
TIMM20122	GCA_032157395.1	India	VIII
TIMM20123	GCA_032157405.1	India	VIII
LL-2024a	SAMN44006357	China	I/II
BE-1	SAMN43365157	Belgium	NA
BE-5	SAMN43365161	Belgium	NA
BE-13	SAMN43365169	Belgium	NA
PG12DES	JBKIZU000000000	Italy	VII

Abbreviations: NA, Not available.

**Table 3:** Characteristics of the *T. mentagrophytes* clinical strains included in this study.

Strain	Species	Clinical Sample	Source	Year of Isolation	*SQLE* Gene	Genotype
ARTRO	*T. verrucosum*	NA	NA	2020	NA	NA
PG10719	*T. mentagrophytes*	Nail scales	Toenails	2020	WT	I/II
PG11516	*T. mentagrophytes*	Nail scales	Big toe	2020	WT	I/II
PG10565	*Beauveria bassiana*	Beard hair	Beard	2020	NA	NA
PG10857	*T. mentagrophytes*	Skin scales	Face	2020	WT	VII
PG10MUR	*T. mentagrophytes*	Nail scales	Toenails	2021	WT	I/II
PG11338	*T. mentagrophytes*	NA	Face	2021	Lys276Asn	I/II
PG11035	*T. mentagrophytes*	Nail scales	Toenails	2022	WT	I/II
PG12DES	*T. mentagrophytes*	Skin scales	Periocular	2022	WT	VII
PG13426	*T. mentagrophytes*	Skin scales	NA	2023	Lys276Asn	I/II
PG9FER	*T. mentagrophytes*	Nail scales	Big toe	2023	WT	I/II
PG13490	*T. mentagrophytes*	Nail scales	Big toe	2024	WT	I/II
PG13334	*T. mentagrophytes*	Nail scales	Thumb	2024	WT	I/II

Abbreviations: NA, Not available; WT, Wild type.

**Table 4:** Genomic characteristics of the *T. mentagrophytes* genomes included in this study.

Genome Characteristics	PG9FER	PG10MUR	PG11338	PG12DES
Total Length (bp)	22,823,954	22,793,562	23,205,410	23,117,013
No. of Contigs	615	679	712	306
N50	91.543	91.906	100.681	160.843
N90	24.082	22.100	24.509	41.955
L50	79	79	71	47
L90	257	275	241	156
G + C (%)	47.98	48	47.52	47.71
No. of predicted genes	7.124	7.140	7.140	7.187

**Table 5:** Aminoacid substitutions on virulence factors within *T. mentagrophytes* genomes included in this study.

Virulence Factors	PG9FER	PG10MUR	PG11338	PG12DES
MEP-1	S62A	G153D	S62A	S62A, G153D
MEP-2	Y334F	Y334F	Y334F	Y334F, N517S
MEP-3	D122G	WT	D122G	I573L, R615K, P620A
MEP-4	NC	NC	NC	WT
MEP-5	N164S, WPI252-254RAL, Del255S, T265S, I510T	H39R, N164S, WPI252-254RAL, Del255S, T265S	N164S, WPI252-254RAL, Del255S, T265S, I510T	NC
ZafA	F111S, Del366-401, D402Y, Ins484N, Del485-505	Del366-401, D402Y, Ins484T	F111S, Del366-401, D402Y, Ins484T	Ins484N

Abbreviations: WT, Wild type; NC, Not counted.

## Data Availability

The draft genome assemblies of *T. mentagrophytes* strains have been deposited in the NCBI under the following accession numbers: JBLIVW000000000 (Biosample: SAMN46524735; PG9FER), JBLIVX000000000 (Biosample: SAMN46524736, PG10MUR), JBLIVY000000000 (Biosample: SAMN46524737, PG11338), and JBKIZU000000000 (Biosample: SAMN45682025, PG12DES).

## Supplementary Materials

The following supporting information can be downloaded at: https://www.mdpi.com/article/10.3390/jof11080566/s1, Figure S1: Aminoacid aligment of virulence factors (MEP-1, panel A; MEP-2, panel B; MEP-3, panel C; MEP-4, panel D; MEP-5, panel E; ZafA, panel F) in the genomes of *T. mentagrophytes* genomes included in this study compared to reference alleles. Aminoacid variations were highlighted.
