# Supplementary material for: Epidemiology and Genomic Characterization of Trichophyton mentagrophytes over a Period of 4 Years in Northern Italy
Source: J Fungi (Basel). 2025 Jul 29;11(8):566. doi: 10.3390/jof11080566 (PMC12387727; doi:10.3390/jof11080566)

Figure S1. Aminoacid aligment of virulence factors (MEP-1, panel A; MEP-2, panel B; MEP-3, panel C; MEP-4, panel D; MEP-5, panel E; ZafA, panel F) in the genomes of *T. mentagrophytes* genomes included in this study compared to reference alleles. Aminoacid variations were highlighted

Panel A

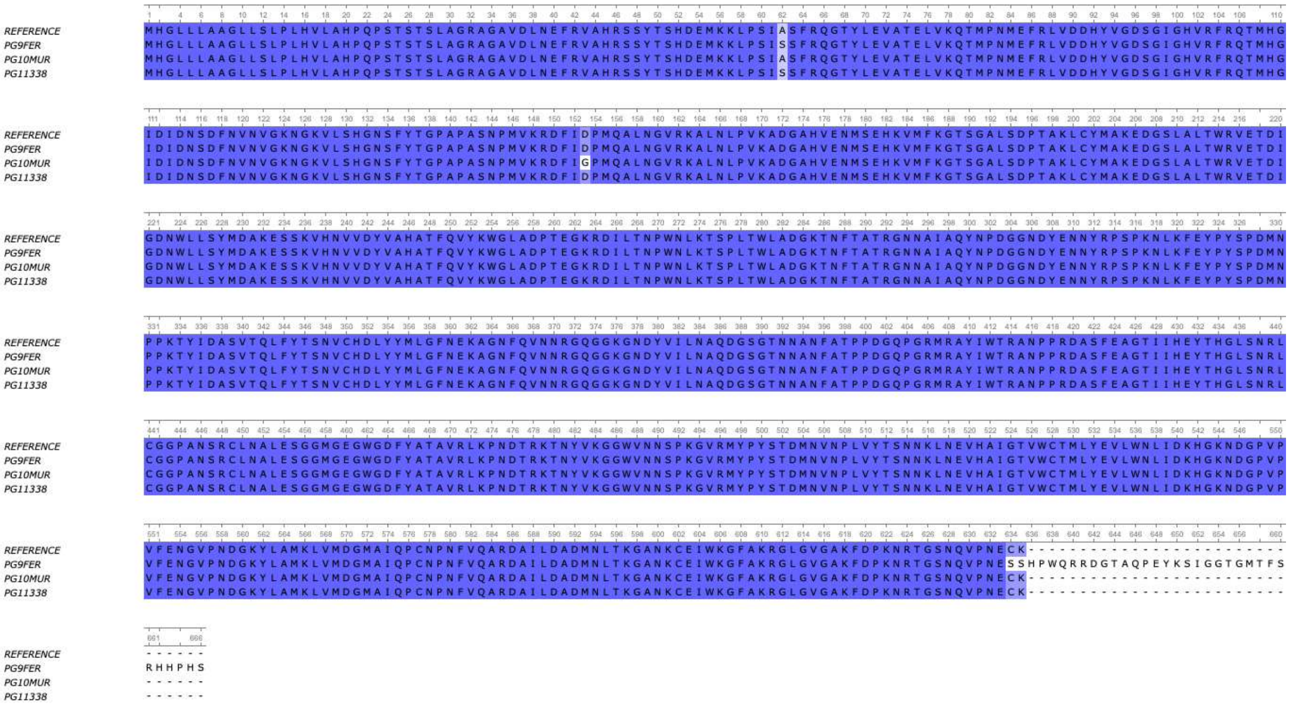

Panel B

```
                1   4   6   8   10  12  14  16  18  20  22  24  26  28  30  32  34  36  38  40  42  44  46  48  50  52  54  56  58  60  62  64  66  68  70  72  74  76  78  80  82  84  86  88  90  92  94  96  98  100  103
REFERENCE       MHGLLLAGLAAALPLGVAGLPARQQSGLSPRGIDINPYRFASMAKYSEHKATSQMVHSFSYSKDDDYVATATKLVKSTFPNMTFRTVKDHYIGTNGIGHVHFK
PG9FER          MHGLLLAGLAAALPLGVAGLPARQQSGLSPRGIDINPYRFASMAKYSEHKATSQMVHSFSYSKDDDYVATATKLVKSTFPNMTFRTVKDHYIGTNGIGHVHFK
PG10MUR         MHGLLLAGLAAALPLGVAGLPARQQSGLSPRGIDINPYRFASMAKYSEHKATSQMVHSFSYSKDDDYVATATKLVKSTFPNMTFRTVKDHYIGTNGIGHVHFK
PG11338         MHGLLLAGLAAALPLGVAGLPARQQSGLSPRGIDINPYRFASMAKYSEHKATSQMVHSFSYSKDDDYVATATKLVKSTFPNMTFRTVKDHYIGTNGIGHVHFK

                104  108 110 112 114 116 118 120 122 124 126 128 130 132 134 136 138 140 142 144 146 148 150 152 154 156 158 160 162 164 166 168 170 172 174 176 178 180 182 184 186 188 190 192 194 196 198 200 202  206
REFERENCE       QTAHGIDIDNADFNVNIGRDGKVFTFGNSFYEGEMPKTNPLTKRDFADPVKALHGAIKTLKLPVKPQSAKAMPMKEAETFMFEGTSGALSEPMAKLVYIQKDG
PG9FER          QTAHGIDIDNADFNVNIGRDGKVFTFGNSFYEGEMPKTNPLTKRDFADPVKALHGAIKTLKLPVKPQSAKAMPMKEAETFMFEGTSGALSEPMAKLVYIQKDG
PG10MUR         QTAHGIDIDNADFNVNIGRDGKVFTFGNSFYEGEMPKTNPLTKRDFADPVKALHGAIKTLKLPVKPQSAKAMPMKEAETFMFEGTSGALSEPMAKLVYIQKDG
PG11338         QTAHGIDIDNADFNVNIGRDGKVFTFGNSFYEGEMPKTNPLTKRDFADPVKALHGAIKTLKLPVKPQSAKAMPMKEAETFMFEGTSGALSEPMAKLVYIQKDG

                207  210 212 214 216 218 220 222 224 226 228 230 232 234 236 238 240 242 244 246 248 250 252 254 256 258 260 262 264 266 268 270 272 274 276 278 280 282 284 286 288 290 292 294 296 298 300 302 304 306  309
REFERENCE       KLHLTWRVETDVGDNWLLSYVDSKETETVHNVVDYVASADYKVFAWGLNDPTEGQPTMIKDPWNTTGSGSPFTWHGDGEMDYTVTRGNNIAAQDNPSGGGQWE
PG9FER          KLHLTWRVETDVGDNWLLSYVDSKETETVHNVVDYVASADYKVFAWGLNDPTEGQPTMIKDPWNTTGSGSPFTWHGDGEMDYTVTRGNNIAAQDNPSGGGQWE
PG10MUR         KLHLTWRVETDVGDNWLLSYVDSKETETVHNVVDYVASADYKVFAWGLNDPTEGQPTMIKDPWNTTGSGSPFTWHGDGEMDYTVTRGNNIAAQDNPSGGGQWE
PG11338         KLHLTWRVETDVGDNWLLSYVDSKETETVHNVVDYVASADYKVFAWGLNDPTEGQPTMIKDPWNTTGSGSPFTWHGDGEMDYTVTRGNNIAAQDNPSGGGQWE

                310  314 316 318 320 322 324 326 328 330 332 334 336 338 340 342 344 346 348 350 352 354 356 358 360 362 364 366 368 370 372 374 376 378 380 382 384 386 388 390 392 394 396 398 400 402 404 406 408  412
REFERENCE       NNYRPESPELSFVYEYNEQMEPEQFKDFAITQLFYTTNTFHDLLYSFGFTEEAGNFQMNNNGKGGEGNDFAICNAQDGSGTNNANFATPPDGQNGRMRMYTWT
PG9FER          NNYRPESPELSFVYEYNEQMEPEQYKDFAITQLFYTTNTFHDLLYSFGFTEEAGNFQMNNNGKGGEGNDFAICNAQDGSGTNNANFATPPDGQNGRMRMYTWT
PG10MUR         NNYRPESPELSFVYEYNEQMEPEQYKDFAITQLFYTTNTFHDLLYSFGFTEEAGNFQMNNNGKGGEGNDFAICNAQDGSGTNNANFATPPDGQNGRMRMYTWT
PG11338         NNYRPESPELSFVYEYNEQMEPEQYKDFAITQLFYTTNTFHDLLYSFGFTEEAGNFQMNNNGKGGEGNDFAICNAQDGSGTNNANFATPPDGQNGRMRMYTWT

                413  416 418 420 422 424 426 428 430 432 434 436 438 440 442 444 446 448 450 452 454 456 458 460 462 464 466 468 470 472 474 476 478 480 482 484 486 488 490 492 494 496 498 500 502 504 506 508 510 512  515
REFERENCE       TAQPSRDGDLEAGIVIHEYAHGLSNRLCGGPANSNCLSELEAGGMGEGWGDFYATAIRLKQDDTRETDYTMGEWAANMEGGIREYPYSTNMQTNPYTYADVEG
PG9FER          TAQPSRDGDLEAGIVIHEYAHGLSNRLCGGPANSNCLSELEAGGMGEGWGDFYATAIRLKQDDTRETDYTMGEWAANMEGGIREYPYSTNMQTNPYTYADVEG
PG10MUR         TAQPSRDGDLEAGIVIHEYAHGLSNRLCGGPANSNCLSELEAGGMGEGWGDFYATAIRLKQDDTRETDYTMGEWAANMEGGIREYPYSTNMQTNPYTYADVEG
PG11338         TAQPSRDGDLEAGIVIHEYAHGLSNRLCGGPANSNCLSELEAGGMGEGWGDFYATAIRLKQDDTRETDYTMGEWAANMEGGIREYPYSTNMQTNPYTYADVEG

                516  520 522 524 526 528 530 532 534 536 538 540 542 544 546 548 550 552 554 556 558 560 562 564 566 568 570 572 574 576 578 580 582 584 586 588 590 592 594 596 598 600 602 604 606 608 610 612 614  618
REFERENCE       MSEVHGIGTVWATILYDVLWNLIDEHGMSKNIMPKFVNGAPSDGRNLAMKLVLDGMTLMPCNPNFVQARDAIIDADQALTNGQNKCALMKAFSKRGLGSNYKH
PG9FER          MSEVHGIGTVWATILYDVLWNLIDEHGMSKNIMPKFVNGAPSDGRNLAMKLVLDGMTLMPCNPNFVQARDAIIDADQALTNGQNKCALMKAFSKRGLGSNYKH
PG10MUR         MSEVHGIGTVWATILYDVLWNLIDEHGMSKNIMPKFVNGAPSDGRNLAMKLVLDGMTLMPCNPNFVQARDAIIDADQALTNGQNKCALMKAFSKRGLGSNYKH
PG11338         MSEVHGIGTVWATILYDVLWNLIDEHGMSKNIMPKFVNGAPSDGRNLAMKLVLDGMTLMPCNPNFVQARDAIIDADQALTNGQNKCALMKAFSKRGLGSNYKH

                619  622 624 626 628  632
REFERENCE       GKNRVNNFDMPADC
PG9FER          GKNRVNNFDMPADC
PG10MUR         GKNRVNNFDMPADC
PG11338         GKNRVNNFDMPADC
```

Panel C

```
          1                                                                                                      104
REFERENCE MHGLLLAGLLALPMNVLAHPAEQHASNVLSRRGVDIESFRLPLKAKYMDSDAAAQKIQAMSFSKDDDYVSTATKLVKSTFPKSTFRVVDDHYIGTNGIGHVHFK
PG9FER    MHGLLLAGLLALPMNVLAHPAEQHASNVLSRRGVDIESFRLPLKAKYMDSDAAAQKIQAMSFSKDDDYVSTATKLVKSTFPKSTFRVVDDHYIGTNGIGHVHFK
PG10MUR   MHGLLLAGLLALPMNVLAHPAEQHASNVLSRRGVDIESFRLPLKAKYMDSDAAAQKIQAMSFSKDDDYVSTATKLVKSTFPKSTFRVVDDHYIGTNGIGHVHFK
PG11338   MHGLLLAGLLALPMNVLAHPAEQHASNVLSRRGVDIESFRLPLKAKYMDSDAAAQKIQAMSFSKDDDYVSTATKLVKSTFPKSTFRVVDDHYIGTNGIGHVHFK

          105                                                                                                    208
REFERENCE QTAHGLDIDNSDFNVNIGRDGKVFSFGNSFFTGEIPKENPMVKRAFSDPVKALKGAVKALNLPVKSDNAKAKTTAGKESFEFMGTTGALSAPKANLVYLQKEDG
PG9FER    QTAHGLDIDNSDFNVNIDRDGKVFSFGNSFFTGEIPKENPMVKRAFSDPVKALKGAVKALNLPVKSDNAKAKTTAGKESFEFMGTTGALSAPKANLVYLQKEDG
PG10MUR   QTAHGLDIDNSDFNVNIGRDGKVFSFGNSFFTGEIPKENPMVKRAFSDPVKALKGAVKALNLPVKSDNAKAKTTAGKESFEFMGTTGALSAPKANLVYLQKEDG
PG11338   QTAHGLDIDNSDFNVNIDRDGKVFSFGNSFFTGEIPKENPMVKRAFSDPVKALKGAVKALNLPVKSDNAKAKTTAGKESFEFMGTTGALSAPKANLVYLQKEDG

          209                                                                                                    312
REFERENCE TLALTWRVETDVGDNWLLTYVDAHNSETVHNVVDYVASAEFKVFAWGLNDPTEGNPTSIRDPWTDSSPYTWHSDGMTKYPTTRGNNAIAQDNPTGGSTYINNYR
PG9FER    TLALTWRVETDVGDNWLLTYVDAHNSETVHNVVDYVASAEFKVFAWGLNDPTEGNPTSIRDPWTDSSPYTWHSDGMTKYPTTRGNNAIAQDNPTGGSTYINNYR
PG10MUR   TLALTWRVETDVGDNWLLTYVDAHNSETVHNVVDYVASAEFKVFAWGLNDPTEGNPTSIRDPWTDSSPYTWHSDGMTKYPTTRGNNAIAQDNPTGGSTYINNYR
PG11338   TLALTWRVETDVGDNWLLTYVDAHNSETVHNVVDYVASAEFKVFAWGLNDPTEGNPTSIRDPWTDSSPYTWHSDGMTKYPTTRGNNAIAQDNPTGGSTYINNYR

          313                                                                                                    416
REFERENCE PQSPNLIFNYPWSPTATPPSSYKDFSITQLFYTTNRFHDLLYSFGFNEAAGNFQVNNGNKGGRGNDFAIVNAQDGSGTNNANFATPPDGSPGRMRMYNWTTARP
PG9FER    PQSPNLIFNYPWSPTATPPSSYKDFSITQLFYTTNRFHDLLYSFGFNEAAGNFQVNNGNKGGRGNDFAIVNAQDGSGTNNANFATPPDGSPGRMRMYNWTTARP
PG10MUR   PQSPNLIFNYPWSPTATPPSSYKDFSITQLFYTTNRFHDLLYSFGFNEAAGNFQVNNGNKGGRGNDFAIVNAQDGSGTNNANFATPPDGSPGRMRMYNWTTARP
PG11338   PQSPNLIFNYPWSPTATPPSSYKDFSITQLFYTTNRFHDLLYSFGFNEAAGNFQVNNGNKGGRGNDFAIVNAQDGSGTNNANFATPPDGSPGRMRMYNWTTARP

          417                                                                                                    520
REFERENCE NRDGCLEAGIVIHEYAHGLSNRLCGGPANSGCLNALESGGMGEGWGDFYATAIRLKPRDTKDTNYSMGAWAANNPKGIRAYLYSTNLQTNPYMYTSVNSLREVH
PG9FER    NRDGCLEAGIVIHEYAHGLSNRLCGGPANSGCLNALESGGMGEGWGDFYATAIRLKPRDTKDTNYSMGAWAANNPKGIRAYLYSTNLQTNPYMYTSVNSLREVH
PG10MUR   NRDGCLEAGIVIHEYAHGLSNRLCGGPANSGCLNALESGGMGEGWGDFYATAIRLKPRDTKDTNYSMGAWAANNPKGIRAYLYSTNLQTNPYMYTSVNSLREVH
PG11338   NRDGCLEAGIVIHEYAHGLSNRLCGGPANSGCLNALESGGMGEGWGDFYATAIRLKPRDTKDTNYSMGAWAANNPKGIRAYLYSTNLQTNPYMYTSVNSLREVH

          521                                                                                                    624
REFERENCE QIGTVWATMLYDLMWALIEAHGGTYSANPVFRNGVPQDGRHLAMKLVMDGMALQPCNPNFVQARDAILDADRALTNSANKCTIWKAFAKRGLGYGAKYDARNRT
PG9FER    QIGTVWATMLYDLMWALIEAHGGTYSANPVFRNGVPQDGRHLAMKLVMDGMALQPCNPNFVQARDAILDADRALTNSANKCTIWKAFAKRGLGYGAKYDARNRT
PG10MUR   QIGTVWATMLYDLMWALIEAHGGTYSANPVFRNGVPQDGRHLAMKLVMDGMALQPCNPNFVQARDAILDADRALTNSANKCTIWKAFAKRGLGYGAKYDARNRT
PG11338   QIGTVWATMLYDLMWALIEAHGGTYSANPVFRNGVPQDGRHLAMKLVMDGMALQPCNPNFVQARDAILDADRALTNSANKCTIWKAFAKRGLGYGAKYDARNRT

          625     633
REFERENCE GSNRLPPGC
PG9FER    GSNRLPPGC
PG10MUR   GSNRLPPGC
PG11338   GSNRLPPGC
```

Panel D

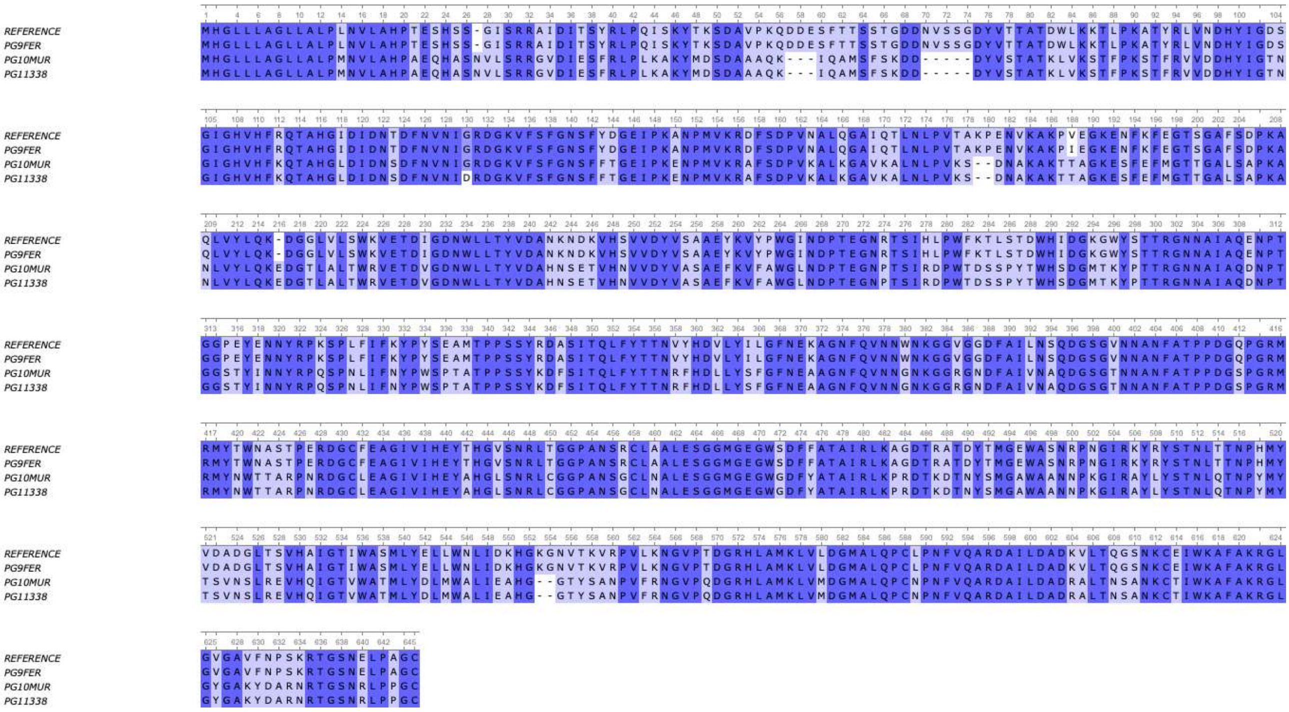

Panel E

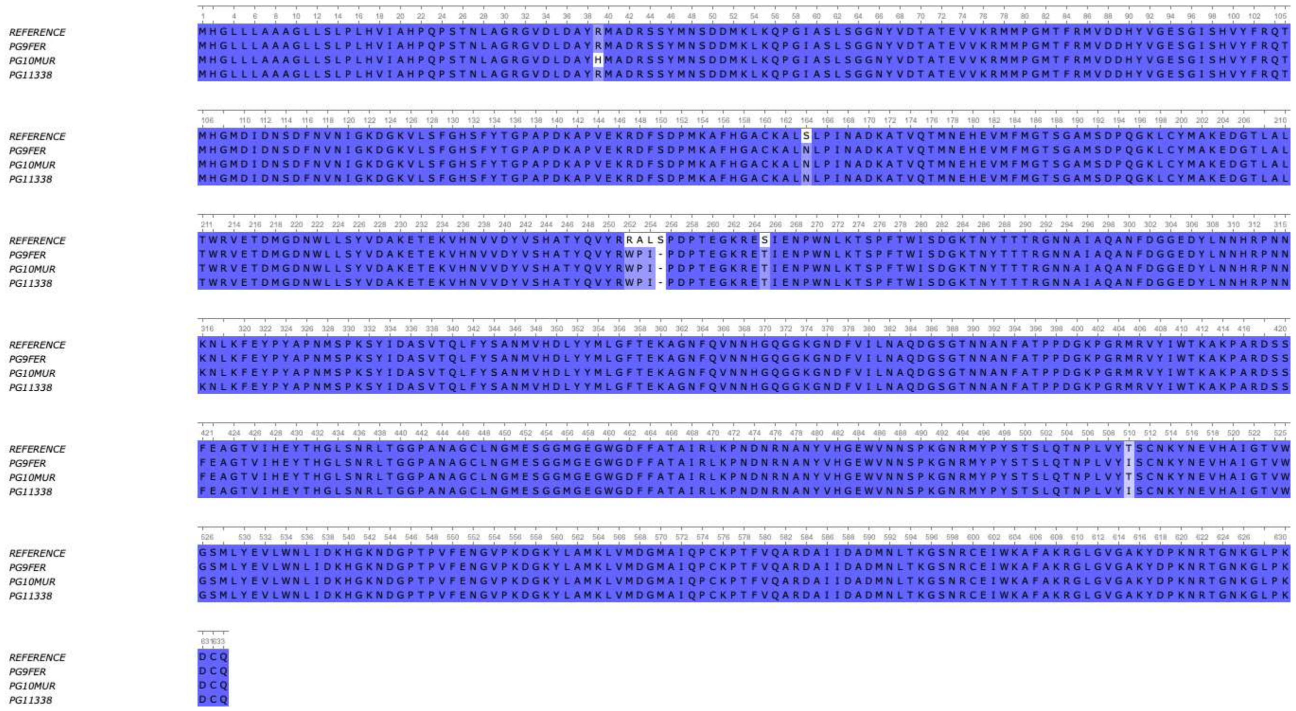

Panel F

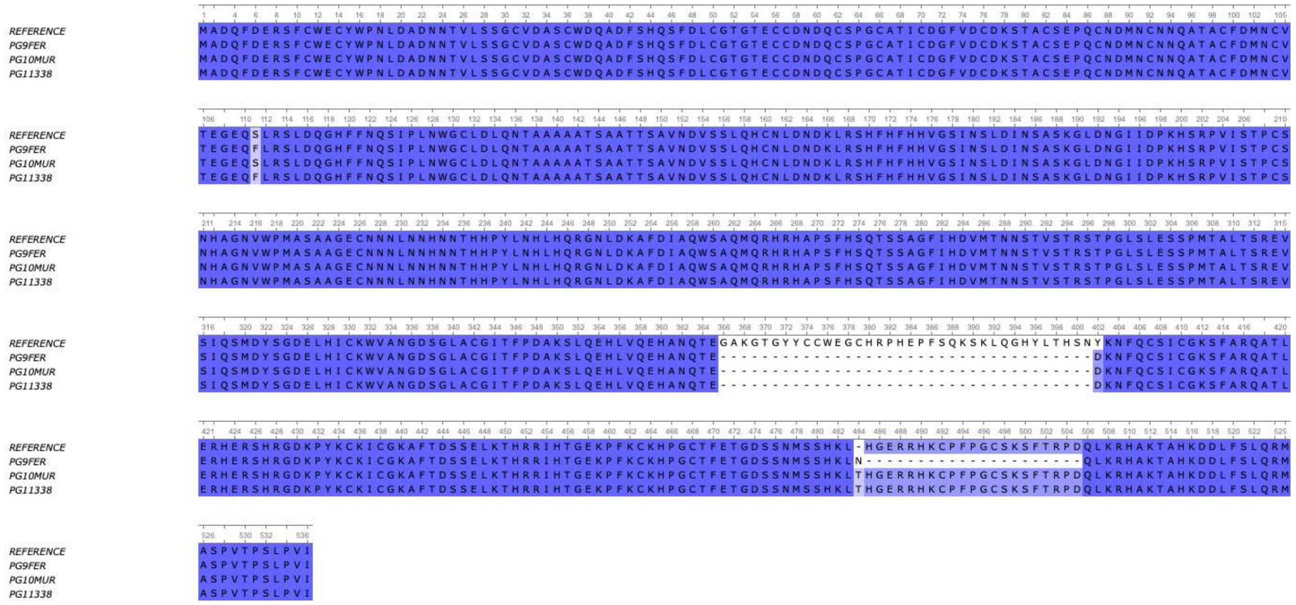

Supplement: Supplementary file 1 [file jof-11-00566-s001.zip › jof-3728403-supplementary.pdf]
